# Prostate-MRI: experience of the observer and technical conditions influence the cancer detection rate

**DOI:** 10.1186/1470-7330-14-S1-P12

**Published:** 2014-10-09

**Authors:** S Roedel, S Blaut, E Duerig, M Burke, R Paulick, G Haroske, F Steinbach, T Kittner

**Affiliations:** 1 Krankenhaus Dresden-Friedrichstadt, Germany

## Aim

The prostate cancer (PCa) detection rate of MR-guided biopsy (MRGB) increased in our hospital from 36.6% in 2012 to 69% in 2013. The study analysed the values of the mpMRI-characteristics of identifiable lesions retrospectively to show the influence of the increasing experience of the observer and modified technical conditions.

## Methods

56 patients (pat.) with mostly at least one prior negative TRUS-guided biopsy and persistent suspicion of PCa with at least one mpMRI-defined identifiable cancer suspicious lesion were included in this study between 2012 and 2013. MpMRI: 1.5 T/e-coil/T2WI/DWI, b-values 2012: 0-1500, 2013: 100-1500/DCE-MRI]. MRGB: in-bore. Characteristics of lesions (ADC, ESUR PIRADS) were statistically correlated with core needle biopsy results (ROC). A p value of p < 0.05 was considered as statistically significant.

## Results

2012/2013: detection rate of all suspicious lesions 33%/58%; in peripheral zone 45%/50%; in transitional zone 14%/67%. The ROC curve area difference was statistically significant for 2012/2013 for ADC 0.65/0.83 (P=0.008). The cut-off values [cut-off (sensitivity; specificity)]: 2012/2013: ADC 836 (0.58;0.58) / 651 (0.72;0.71); 2013: PIRADS DWI 3.5 (0.57;1.0), PIRADS DCE 3.5 (0.63;0.69), PIRADS T2 3.5 (0.71;0.86).

## Conclusion

Modified DWI as to exclude microcapillary perfusion effects leads to lower cut-off value and higher diagnostic value of the ADC. The increasing experience of the observer enhances the evaluation of the transitional zone. The combination of the modified technical conditions and increasing experience of the observer leads to higher sensitivity and specificity of the overall mpMRI prostate evaluation and (PCa) detection rate.

